# Evaluation of efficacy and safety of Reteplase and Alteplase in the treatment of hyper-acute cerebral infarction

**DOI:** 10.1042/BSR20170730

**Published:** 2018-01-17

**Authors:** Zhi-Jian Lin, Hong-Yan Qiu, Xiao-Xin Tong, Yi Guo, Man-Fu Han, Chun-Shui Yang, Kai-Hua Lin, Jun Wu, Xing Li, Yang Yang

**Affiliations:** 1 Department of Neurology, Peking University Shenzhen Hospital, Shenzhen 518036, P.R. China; 2 Department of Neurology, Shenzhen People's Hospital, Shenzhen 518020, P.R. China; 3 Department of Neurology, Shenzhen Second People's Hospital, Shenzhen 518305, P.R. China; 4 Department of Neurology, Shenzhen Sixth People's Hospital, Shenzhen 518052, P.R. China; 5 Department of Clinical Medicine, Guangdong Medical University, Zhanjiang 524023, P.R. China

**Keywords:** Alteplase, Efficacy, Hyper-acute cerebral infarction, Reteplase, Safety

## Abstract

**Objective:** The present study aimed to investigate the efficacy and safety of Reteplase (rPA) and Alteplase (rt-PA) in the treatment of hyper-acute cerebral infarction (CI).

**Methods:** Six hundred and eleven patients with hyper-acute CI selected from September 2014 to September 2016 were assigned into the aspirin, rt-PA, rPA, rt-PA + aspirin, and rPA + aspirin groups based on their willingness. The difference of efficacy in five groups were evaluated with National Institute of Health Stroke Scale (NIHSS), modified rankin scale (mRS), and Barthel Index (BI). Coagulation function, blood lipid, and hemodynamics were analyzed. The safety differences were compared by observing the adverse reactions.

**Results:** Compared with the rt-PA, rPA, and aspirin groups, NIHSS score, mRS score, the incidence of non- and symptomatic cerebral hemorrhage as well as the rate of adverse reactions were decreased, while BI were increased in the rt-PA + aspirin and rPA + aspirin groups after treatment. Compared with the rt-PA and rPA groups, total cholesterol (TC), triacylglycerol (TG), low-density lipoprotein cholesterol (LDL-C), and high-density lipoprotein cholesterol (HDL-C) levels were lower, whereas the hematocrit, whole blood high shear viscosity, whole blood low shear viscosity, plasma viscosity, erythrocyte electrophoresis time, fibrinogen, erythrocyte sedimentation rate (ESR), *K* value in blood sedimentation equation, and the comprehensive abnormality degree of blood rheology were higher in the rt-PA + aspirin and rPA + aspirin groups.

**Conclusion:** The efficacy and safety of rt-PA or rPA combined with aspirin in the treatment of hyper-acute CI were better than those of rPA or rt-PA monotherapy.

## Introduction

Cerebral infarction (CI) is a common clinical cerebrovascular disease that is caused by arterial occlusion that blocks blood supply to the brain [1,2]. CI is the most severe subtype of ischemic stroke and it occurs frequently in daily practice [3]. Patients with acute CI suffer a lot, and their falling health is associated with factors such as gender, age, stoke severity, and dementia [4]. Intravenous tissue-type plasminogen activator (t-PA) is an effective early treatment for acute CI if the treatment is given within 3 h after the symptoms [5]. For hyper-acute CI, since its effective treating time is limited within 6 h after the ischemia, it is important to diagnose and predict the causes timely, or the best time for treatment will be delayed [6]. Methods like CT, MRI, and transcranial Doppler are used for diagnosis, but they are usually not effective for they are complex and consumable [7,8]. Therefore, more studies should be done to find better treatments for hyper-acute CI.

Low-dose aspirin is used as a preventive treatment for ischemic heart disease and ischemic cerebrovascular disease, on the other hand, gastrointestinal injuries are an adverse effect of aspirin [9]. Some data showed that combination therapy with clopidogrel and aspirin is more effective than aspirin alone in reducing microembolic signals in patients with predominantly intracranial symptomatic stenosis [10]. Both as FDA-approved methods, recombinant tissue plasminogen activator (rt-PA, Alteplase) has been used to treat acute ischemic stroke (AIS) while recombinant plasminogen activator (rPA, Reteplase) has been especially used to treat acute myocardial infarction (AMI) in adults [11,12]. rt-PA is effective to prevent ischemic stroke patients from disability if given within the time window of 4.5 h, or it would be much less effective [13]. For patients receiving hemodialysis, rt-PA can be used to prevent dialysis catheter malfunction and bacteremia as a locking solution, and has better effect than heparin [14]. However, researches find that rt-PA may cause hemorrhagic complication in patients, making its use more limited [15]. As a recombinant thrombolytic peptide, r-PA is a structurally modified type of native t-PA, thus has better qualities in half-life and fibrin specificity and play an important role in the treatment [16]. Besides, it has lower side effects and is easier for administration, and has good effects in treatments for diseases like heart and brain occlusive disorders [17]. In a research about cerebral embolic rabbits, rPA is found to improve the return of cerebral perfusion faster than rtPA [15], but the effectiveness and safety of rRA for the treatment of CI in humans remain to be studied. Therefore, the present study aims to investigate the efficacy and safety of Alteplase and Reteplase used together with aspirin, thus making contribution to the treatment for hyper-acute CI.

## Materials and methods

### Ethics statement

Data collection for the present study was in line with the diagnostic criteria emendatory in the 4th cerebrovascular disease academic conference. The study was based on the conduct norms and standards of the new technology in Peking University Shenzhen Hospital and approved by the medical ethics committee. Written informed consents were obtained from patients (when patients had no capacity for civil conduct, their families would sign the consents after signing attorney letter).

### Study subjects

Six hundred and eleven consecutive patients admitted to neurology department of Peking University Shenzhen Hospital, Shenzhen People's Hospital, Shenzhen Second People's Hospital, and Shenzhen Sixth People's Hospital between September 2014 and September 2016 after diagnosis of hyper-acute CI were included. After communicating with patients and their families about the conditions and regimens, all patients were classified into the aspirin group (*n* = 117), the rt-PA group (*n* = 121), the rPA group (*n* = 123), the rt-PA + aspirin group (*n* = 126), and the rPA + aspirin group (*n* = 124) based on their willingness. The hyper-acute CI patients with thrombolytic indications were treated with intravenous thrombolytic therapy and those who are willing to receive conventional drug therapy were selected into the aspirin group. The inclusion criteria were (1) patients suffered from AIS with no coma; (2) disease occurred within 4.5 h; (3) low-density ischemic foci was not observed via head computerized tomographic (CT) scanning with cerebral hemorrhage eliminated; and (4) age ranked from 18 to 80 years old. The exclusion criteria were (1) patients with single attack of transient ischemic attack (TIA), rapid improvement of stroke and mild symptoms; (2) patients with subarachnoid hemorrhage via medical history and physical examination; (3) patients’ blood pressure was more than 185/110 mmHg after hypotensive therapy for twice; (4) patients showed hemorrhage, brain edema, occupying effect, tumor, and arteriovenous malformation via CT; (5) patients suffered from major surgery or trauma within 14 days, arterial puncture within 7 days, and active continuing hemorrhage; (6) patients were using anticoagulant or using heparin within 48 h before stroke; and (7) patients with blood system diseases, hemorrhagic diathesis, and coagulation disorder. Patients receiving conventional drug therapy (from the aspirin group) were all diagnosed with hyper-acute CI and the indication was consistent with that for thrombolytic therapy. Due to patients’ and their families’ incomplete awareness for hyper-acute CI, economic condition and excessive worry about the complications, patients and their families disagreed with thrombolytic therapy, thus the conventional drug therapy was conducted.

### Treatments

Five groups of patients were treated with sedation, analgesia and other conventional symptomatic treatment, patients in the aspirin group were treated with free radical scavenger, platelet inhibitors, and oral enteric coated aspirin (300 mg for the first time, then 100 mg for each time) (Bayer healthcare Co., Ltd., Beijing, China). Patients in the rt-PA group were treated with intravenous rt-PA thrombolytic therapy (Boehringer Mannheim GmbH). The total dose was 0.6–0.9 mg/kg, 10% of the dose given was administered by peripheral vein (PV) injection within 1 min, the residual dose was administered via venous pump in the subsequent 60 min. The rPA group was treated with rPA (Shandong Colla Asini incorporated company, Shangdong, China) intravenous thrombolytic therapy, for the first time, 10% of the total rPA was mixed with 10 ml saline in the syringe for 3 min of rapid intravenous injection, then the remaining 90% rPA was followed by adding 100 ml of saline with intravenous pump, and the thrombolytic time was within 1 h. The rPA + aspirin group were treated with rPA combined with aspirin, the rt-PA + aspirin group were treated with rt-PA combined with aspirin, the specific dose was the same as the above. The vital signs of the patients were closely monitored during the medication, and the patients were according to the above-mentioned mode of administration for 4 weeks.

### Therapeutic evaluation

The efficacy of each group was evaluated by comparing the National Institute of Health Stroke Scale (NIHSS) [18], modified rankin scale (mRS) [19], and Barthel Index (BI) [20] before the treatment and 30 days after treatment. NIHSS scoring criteria were basic recovery (NIHSS decreased by 90–100%); significant progress (NIHSS decreased by 46–90%); progress (NIHSS decreased by 18–45%), no changes (NIHSS decreased less than 18%); deterioration (NIHSS after treatment > NIHSS before treatment); and death. The mRS scoring criteria were as follows: normal (0–1 point), recovery of the self-care ability (1–2 points), moderate neurological dysfunction (2–3 points), and severe neurological dysfunction (4–5 points). The BI score items include 10 aspects, such as eating, bathing, dressing, walking, toileting, etc. Scores of 61–99 points were taken as mild functional independence impairment, scores of 41–60 points were regarded as moderate functional independence impairment, and scores ≤ 40 points were considered as severe functional independence impairment.

The prothrombin time (PT), activated partial thromboplastin time (APTT), human fibrinogen (FIB), total cholesterol (TC), triacylglycerol (TG), high-density lipoprotein cholesterol (HDL-C), and low-density lipoprotein cholesterol (LDL-C) were detected by OLPMPUS automatic biochemical analyzer (Wenzhou Dongou Biological Engineering Co. Ltd., Wenzhou, Zhejiang, China) before and after treatment. Hematocrit, whole blood high shear viscosity, whole blood low shear viscosity, plasma viscosity, erythrocyte electrophoretic time, fibrinogen, erythrocyte sedimentation rate (ESR), *K* value in blood sedimentation equation, and the comprehensive abnormality degree of blood rheology were observed and recorded before treatment and 30 days after treatment.

### Safety evaluation and follow-up

Thirty days after treatment, the recurrence rate of CI, the incidence of symptomatic cerebral hemorrhage, non-symptomatic cerebral hemorrhage, and mortality were observed to evaluate the safety. After treatment and agreed by themselves and their families, the patients were regularly consulted by telephone, outpatient follow-up, petition, or access to case data to do the follow-up. The follow-up time was 6 months, and the follow-up completion rate was 100%. The patient's condition changes were observed and the incidence of adverse reactions in each group was analyzed through regular follow-up feedback.

### Statistical analysis

SPSS 20.0 statistical software (New York, IL, USA) was used for statistical analysis of all the data. The data of the numerical variables between the groups were in normal distribution, and the measurement data were expressed as mean ± standard deviation (SD). The differences among the groups were analyzed by variance analysis. The difference was significant if *P* < 0.05.

## Results

### Baseline characteristics of patients in each group

As shown in Table 1, there was no significant difference among the aspirin, rt-PA, rPA, rt-PA + aspirin, and rPA + aspirin groups in the aspect of age, gender, history of hypertension, history of diabetes, etc. (all *P* > 0.05), indicating that the patients were comparable.

**Table 1:** Baseline characteristics among the aspirin, rt-PA, rPA, rt-PA + aspirin, and rPA + aspirin groups

Characteristics	Aspirin group (*n* = 117)	rt-PA group (*n* = 121)	rPA group (*n* = 123)	rt-PA + aspirin group (*n* = 126)	rPA + aspirin group (*n* = 124)	*P*
Age (years)	60.82 ± 10.17	62.02 ± 8.76	59.72 ± 11.53	61.85 ± 9.69	62.98 ± 7.83	0.072
Gender (male/female)	61/56	67/54	80/43	68/58	64/60	0.203
History of hypertension (*n*)	47	50	51	53	64	0.355
History of diabetes (*n*)	35	36	37	38	46	0.676
History of coronary heart disease (*n*)	58	61	63	65	51	0.44
History of atrial fibrillation (*n*)	22	24	25	27	33	0.604
History of smoking (*n*)	60	63	65	66	73	0.763
History of drinking (*n*)	18	21	22	24	29	0.583
History of stroke (*n*)	24	26	29	34	31	0.691
Systolic pressure (mmHg)	153.86 ± 21.34	154.93 ± 22.18	155.68 ± 25.19	157.60 ± 21.20	157.01 ± 21.56	0.775
Diastolic pressure (mmHg)	87.78 ± 15.23	88.06 ± 15.74	88.43 ± 14.92	89.93 ± 15.16	90.17 ± 14.16	0.624
Blood glucose levels (mmol/l)	6.26 ± 2.38	6.57 ± 2.31	6.52 ± 2.22	6.99 ± 2.33	6.86 ± 2.43	0.115
Duration from the occurrence to the therapy (h)	3.48 ± 1.68	3.49 ± 1.67	3.45 ± 1.80	3.87 ± 1.65	3.73 ± 1.70	0.206

Notes: rPA, Reteplase; rt-PA, Alteplase.

### rt-PA or rPA combined with aspirin had better NIHSS score, mRS score, and BI in the treatment of hyper-acute CI

Table 2 shows that the NIHSS score and mRS score after 30 days of treatment had decreased in the aspirin, rt-PA, rPA, rt-PA + aspirin, and rPA + aspirin groups when compare with that before treatment (all *P* < 0.05). In the rt-PA + aspirin group and the rPA + aspirin group, the NIHSS score and mRS score were obviously better than that in the aspirin, rt-PA, and rPA groups (all *P* < 0.05). But there was no significant difference between the rt-PA + aspirin group and the rPA + aspirin group (*P* > 0.05). Thirty days after treatment, in the aspect of BI, the rt-PA + aspirin and rPA + aspirin groups were better than the aspirin, rt-PA, and rPA groups (all *P* < 0.05), while the rt-PA + aspirin and rPA + aspirin groups had no significant difference (*P* > 0.05) (Table 3). The results suggested that rt-PA or rPA combined with aspirin had better NIHSS score, mRS score, and BI in the treatment of hyper-acute CI.

**Table 2:** NIHSS and mRS scores among the aspirin, rt-PA, rPA, rt-PA + aspirin, and rPA + aspirin groups before and after treatment

Groups	Time	NIHSS score	mRS score
Aspirin group (*n* = 117)	Before treatment	13.31 ± 3.11	4.61 ± 0.57
	30 days after treatment	6.49 ± 1.16^1^	3.12 ± 0.59^1^
rt-PA group (*n* = 121)	Before treatment	13.67 ± 6.22	4.62 ± 0.61
	30 days after treatment	4.85 ± 1.6^1^	3.18 ± 0.61^1^
rPA group (*n* = 123)	Before treatment	13.61 ± 6.08	4.64 ± 0.56
	30 days after treatment	4.44 ± 2.06^1^	3.21 ± 0.57^1^
rt-PA + aspirin group (*n* = 126)	Before treatment	13.37 ± 5.31	4.63 ± 0.52
	30 days after treatment	3.16 ± 0.56^1,2^	2.13 ± 0.48^1,2^
rPA + aspirin group (*n* = 124)	Before treatment	13.34 ± 5.12	4.65 ± 0.54
	30 days after treatment	3.15 ± 0.54^1,2,3^	2.14 ± 0.56^1,2,3^

Notes: rPA, Reteplase; rt-PA, Alteplase.^1^*P* < 0.05, compared with the same group before treatment.^2^*P* < 0.05, compared with the aspirin, rt-PA, rPA groups at the same time.^3^*P* > 0.05, compared with the rt-PA + aspirin group at the same time.

**Table 3:** BI after 30 days of treatment among the aspirin, rt-PA, rPA, rt-PA + aspirin, and rPA + aspirin groups (*n*, %)

Groups	61–99 points	41–60 points	≤40 points
Aspirin group (*n* = 117)	55 (47.01)	42 (35.90)	20 (17.09)
rt-PA group (*n* = 121)	65 (53.72)	39 (32.23)	17 (14.05)
rPA group (*n* = 123)	64 (52.03)	40 (32.52)	19 (15.45)
rt-PA + aspirin group (*n* = 126)	85 (67.46)^1^	32 (25.4)	9 (7.14)
rPA + aspirin group (*n* = 124)	83 (66.94)^1,2^	31 (25.0)	10 (8.06)

Notes: rPA, Reteplase; rt-PA, Alteplase.^1^*P* < 0.05, compared with the aspirin, rt-PA, and rPA groups at the same scoring interval. ^2^*P* > 0.05, compared with the rt-PA + aspirin group at the same scoring interval.

### rt-PA or rPA combined with aspirin had better evaluation of coagulation function in the treatment of hyper-acute CI

After treatment, PT and APTT were prolonged, and the FIB decreased in the aspirin, rt-PA, rPA, rt-PA + aspirin, and rPA + aspirin groups (all *P* < 0.05). Compared with the aspirin, rt-PA and rPA groups, PT and APTT in the rt-PA + aspirin group and the rPA + aspirin group were significantly prolonged after treatment, while the FIB significantly decreased (all *P* < 0.05) (Table 4). These findings suggested that rt-PA or rPA combined with aspirin had better evaluation of coagulation function in the treatment of hyper-acute CI.

**Table 4:** Evaluation of coagulation function in the aspirin, rt-PA, rPA, rt-PA + aspirin, and rPA + aspirin groups

Coagulation function	Time	Aspirin group (*n* = 117)	rt-PA group (*n* = 121)	rPA group (*n* = 123)	rt-PA + aspirin group (*n* = 126)	rPA + aspirin group (*n* = 124)
PT (t/s)	Before treatment	11.7 ± 3.6	11.98 ± 3.54	11.8 ± 3.72	11.9 ± 3.9	12.0 ± 3.7
	30 days after treatment	13.45 ± 3.21^1^	13.34 ± 3.52^1^	13.40 ± 3.38^1^	15.4 ± 3.67^1,2^	15.6 ± 3.70^1,2^
APTT (t/s)	Before treatment	29.32 ± 3.42	30.07 ± 3.35	29.3 ± 3.45	29.34 ± 3.6	29.41 ± 3.32
	30 days after treatment	36.41 ± 2.5^1^	37.51 ± 2.12^1^	37.55 ± 2.81^1^	39.8 ± 2.8^1,2^	39.9 ± 3.6^1,2^
FIB (p/g·L-1)	Before treatment	3.78 ± 1.64	3.80 ± 1.52	3.77 ± 1.57	3.76 ± 1.62	3.81 ± 1.59
	30 days after treatment	1.95 ± 0.5^1^	1.93 ± 0.69^1^	1.98 ± 0.69^1^	1.54 ± 0.77^1,2^	1.54 ± 0.74^1,2^

Notes: rPA, Reteplase; rt-PA, Alteplase.^1^*P* < 0.05, compared with the same group before treatment. ^2^*P* < 0.05, compared with the aspirin, rt-PA, rPA groups at the same time.

### rt-PA or rPA combined with aspirin had better assessment of blood lipid changes in the treatment of hyper-acute CI

Before treatment, there was no significant difference in the levels of TC, TG, LDL-C, and HDL-C among the aspirin, rt-PA, rPA, rt-PA + aspirin, and rPA + aspirin groups (all *P* > 0.05). After 30 days of treatment, the levels of TC, TG, LDL-C, and HDL-C in the rPA group, the rt-PA group, the rPA + aspirin group, and the rt-PA + aspirin group were significantly lower than those in the aspirin group (all *P* < 0.05). And the levels of TC, TG, LDL-C, and HDL-C in the rt-PA + aspirin group and the rPA + aspirin group were also significantly decreased as compared with the rt-PA group and the rPA group (all *P* < 0.05). The TC, TG, LDL-C, and HDL-C levels of patients in each group were significantly declined before and after treatment (all *P* < 0.05) (Table 5). These findings revealed that rt-PA or rPA combined with aspirin had better assessment of blood lipid changes in the treatment of hyper-acute CI.

**Table 5:** Changes of blood lipid in the aspirin, rt-PA, rPA, rt-PA + aspirin, and rPA + aspirin groups

Blood lipid index	Aspirin group (*n* = 117)		rt-PA group (*n* = 121)		rPA group (*n* = 123)		rt-PA + aspirin group (*n* = 126)		rPA + aspirin group (*n* = 124)	
	Before treatment	30 days after treatment	Before treatment	30 days after treatment	Before treatment	30 days after treatment	Before treatment	30 days after treatment	Before treatment	30 days after treatment
TC (mmol/l)	4.70 ± 0.41	4.47 ± 0.31^1^	4.72 ± 0.37	3.80 ± 0.38^1,2^	4.71 ± 0.40	3.79 ± 0.36^1,2^	4.16 ± 0.39	3.13 ± 0.34^1,2,3^	4.18 ± 0.35	3.11 ± 0.35^1,2,3^
TG (mmol/l)	1.40 ± 0.22	1.27 ± 0.20^1^	1.43 ± 0.25	1.06 ± 0.24^1,2^	1.42 ± 0.23	1.05 ± 0.27^1,2^	1.13 ± 0.24	0.87 ± 0.21^1,2,3^	1.11 ± 0.23	0.88 ± 0.22^1,2,3^
LDL-C (mmol/l)	3.16 ± 0.29	3.01 ± 0.29^1^	3.15 ± 0.32	2.46 ± 0.23^1,2^	3.14 ± 0.31	2.45 ± 0.24^1,2^	2.56 ± 0.31	2.01 ± 0.20^1,2,3^	2.54 ± 0.30	2.02 ± 0.19^1,2,3^
HDL-C (mmol/l)	1.48 ± 0.15	1.30 ± 0.13^1^	1.47 ± 0.18	1.11 ± 0.15^1,2^	1.45 ± 0.19	1.10 ± 0.14^1,2^	1.27 ± 0.15	0.87 ± 0.14^1,2,3^	1.25 ± 0.17	0.90 ± 0.13^1,2,3^

Notes: rPA, Reteplase; rt-PA, Alteplase.^^1^^*P* < 0.05, compared with the same group before treatment. ^2^*P* < 0.05, compared with the aspirin group at the same time. ^3^*P* < 0.05, compared with the rt-PA and rPA groups at the same time.

### rt-PA or rPA combined with aspirin had better hemodynamics in the treatment of hyper-acute CI

As shown in Table 6, there were significant differences among the aspirin, rt-PA, rPA, rt-PA + aspirin, and rPA + aspirin groups before and after treatment (all *P* < 0.05). In the rPA + aspirin and rt-PA + aspirin groups, the hematocrit, whole blood high shear viscosity, whole blood low shear viscosity, plasma viscosity, erythrocyte electrophoresis time, fibrinogen, ESR, *K* value in blood sedimentation equation, and the comprehensive abnormality degree of blood rheology after treatment were higher than that of the rt-PA group and the rPA group (all *P* < 0.05), and those in the rt-PA group and the rPA group were obviously higher than that in the aspirin group (all *P* < 0.05). It could be concluded that rt-PA or rPA combined with aspirin had better hemodynamics in the treatment of hyper-acute CI.

**Table 6:** The hemodynamic indexes before and after treatment among the aspirin, rt-PA, rPA, rt-PA + aspirin, and rPA + aspirin groups

Hemodynamic index	Aspirin group (*n* = 117)		rt-PA group (*n* = 121)		rPA group (*n* = 123)		rt-PA + aspirin group (*n* = 126)		rPA + aspirin group (*n* = 124)	
	Before treatment	30 days after treatment	Before treatment	30 days after treatment	Before treatment	30 days after treatment	Before treatment	30 days after treatment	Before treatment	30 days after treatment
Hematocrit (l/l)	0.41 ± 0.12^1^	0.47 ± 0.03^2^	0.42 ± 0.14^3^	0.51 ± 0.07^2^	0.42 ± 0.13^3^	0.52 ± 0.08^2^	0.44 ± 0.13^1^	0.58 ± 0.03^2^	0.42 ± 0.11	0.59 ± 0.09^2^
Whole high blood viscosity (mPa s)	5.95 ± 0.72^1^	6.74 ± 0.48^2^	5.96 ± 0.74^3^	7.48 ± 0.68^2^	5.93 ± 0.75^3^	7.49 ± 0.69^2^	5.94 ± 0.73^1^	7.97 ± 0.61^2^	5.93 ± 0.71	7.98 ± 0.63^2^
Whole blood low shear viscosity (mPa s)	10.26 ± 2.47^1^	13.74 ± 1.37^2^	10.28 ± 2.54^3^	14.49 ± 2.49^2^	10.27 ± 2.35^3^	14.47 ± 2.75^2^	10.26 ± 2.33^1^	15.28 ± 2.83^2^	10.29 ± 2.31	15.30 ± 2.76^2^
Plasma viscosity (%)	1.64 ± 0.57^1^	2.04 ± 0.20^2^	2.01 ± 1.22^3^	2.56 ± 1.03^2^	2.08 ± 1.11^3^	2.55 ± 0.97^2^	2.00 ± 1.09^1^	2.92 ± 1.49^c^	2.04 ±1.22	2.92 ± 1.17^2^
Erythrocyte electrophoresis time (s)	15.10 ± 0.56^1^	16.70 ± 0.26^2^	15.13 ± 0.48^3^	17.22 ± 0.84^2^	15.12 ± 0.67^3^	17.23 ± 0.37^2^	15.11 ± 0.62^1^	17.92 ± 0.60^c^	15.12 ± 0.33	17.90 ± 0.68^2^
Fibrinogen (g/l)	3.30 ± 1.18^1^	3.68 ± 1.17^2^	3.59 ± 1.11^3^	4.22 ± 1.14^2^	3.58 ± 1.13^3^	4.20 ± 1.10^2^	3.61 ± 1.11^1^	4.67 ± 1.10^2^	3.58 ± 1.09	4.70 ± 1.30^2^
ESR (mm/h)	9.84 ± 1.53^1^	10.39 ± 1.43^2^	10.57 ± 1.06^3^	10.97 ± 1.88^2^	10.47 ± 1.21	11.04 ± 1.29^2,3^	10.5 ± 1.2^1^	11.56 ± 1.36^2^	10.47 ± 1.18	11.72 ±1.07^2^
*K* value of ESR equation	23.11 ± 0.56^1^	23.63 ± 0.37^2^	23.09 ± 0.48^3^	24.27 ± 0.35^2^	23.10 ± 0.60^3^	24.28 ± 0.40^2^	23.13 ± 0.32^1^	25.13 ± 0.45^2^	23.12 ± 0.56	25.10 ± 0.41^2^
Hemorheology abnormality	64.27 ± 0.65^1^	71.18 ± 0.71^2^	65.28 ± 0.64^3^	71.73 ± 0.1^2^	64.29 ± 0.64^3^	71.69 ± 0.74^2^	67.30 ± 0.61^1^	72.40 ± 0.42^2^	64.29 ± 0.30	71.38 ± 0.65^2^

Notes: rPA, Reteplase; rt-PA, Alteplase. ^1^*P* < 0.05, compared with the rt-PA and rPA groups at the same time. ^2^*P* < 0.05 compared with the same group before treatment.^3^*P* < 0.05, compared with the aspirin group at the same time.

### rt-PA or rPA combined with aspirin had better safety evaluation in the treatment of hyper-acute CI

As shown in Table 7, after 30 days of treatment, the recurrence rate of CI and mortality among the aspirin, rt-PA, rPA, rt-PA + aspirin, and rPA + aspirin groups was not significantly different (all *P* > 0.05). The incidence of symptomatic cerebral hemorrhage in the rt-PA, rPA, rt-PA + aspirin, and rPA + aspirin groups was significantly lower than that in the aspirin group (all *P* < 0.05). The rt-PA + aspirin group and rPA + aspirin group also had lower incidence of symptomatic cerebral hemorrhage when compared with the rt-PA group and the rPA group (all *P* < 0.05). Compared with the aspirin group, the incidence of non-symptomatic cerebral hemorrhage was significantly reduced in the rt-PA, rPA, rt-PA + aspirin, and rPA + aspirin groups (all *P* < 0.05). The rt-PA + aspirin and rPA + aspirin groups also had lower incidence of non-symptomatic cerebral hemorrhage when compared with the rt-PA and rPA groups (all *P* < 0.05). We can conclude that rt-PA or rPA combined with aspirin had better safety evaluation in the treatment of hyper-acute CI.

**Table 7:** Safety evaluation on the patients in the aspirin, rt-PA, rPA, rt-PA + aspirin, and rPA + aspirin groups after 30 days of treatment (%)

Group	Recurrence rate of CI	Incidence of symptomatic cerebral hemorrhage	Non-symptomatic cerebral hemorrhage	Mortality
Aspirin group (*n* = 117)	6 (5.13)^1^	16 (13.68)	18 (15.38)	3 (2.56)^1^
rt-PA group (*n* = 121)	4 (3.31)^1^	10 (8.26)^2^	12 (9.92)^3^	1 (0.83)^1^
rPA group (*n* = 123)	5 (4.07)^1^	9 (7.32)^2^	13 (10.57)^3^	1 (0.81)^1^
rt-PA + aspirin group (*n* = 126)	2 (1.59)^1^	0 (0)^2,5^	3 (2.38)^3,6^	0 (0.00)^1^
rPA + aspirin group (*n* = 124)	1 (0.81)^1^	1 (0.81)^2,5^	2 (1.61)^3,6^	0 (0.00)^1^

Notes: rPA, Reteplase; rt-PA, Alteplase.^1^*P* > 0.05. Comparison of rate of symptomatic cerebral hemorrhage: ^2^*P* > 0.05, compared with the aspirin group; ^5^*P* < 0.05, compared with the rt-PA and rPA groups. Comparison of rate of non-symptomatic cerebral hemorrhage: ^3^*P* > 0.05, compared with the aspirin group; ^6^*P* < 0.05, compared with the rt-PA and rPA groups.

### rt-PA or rPA combined with aspirin had lower adverse reactions in the treatment of hyper-acute CI

During the follow-up period of 6 months, 97 adverse reactions were recorded in all cases, including in the aspirin group (*n* = 17), in the rt-PA group (*n* = 18), in the rPA group (*n* = 20), in the rt-PA + aspirin group (*n* = 22), and in the rPA + aspirin group (*n* = 20). There was no significant difference in the rate of adverse reactions (*P* > 0.05) among the aspirin group (14.53%), the rt-PA group (14.88%), the rPA group (16.26%), the rt-PA + aspirin group (17.46%), and the rPA + aspirin group (16.13%). Adverse reactions in all parts of the bleeding were the most common. The gingival bleeding rate in the aspirin group, the rt-PA group, and the rPA group was higher than that in the rt-PA + aspirin and rPA + aspirin groups (*P* < 0.05). There was no significant difference in the incidence of other adverse reactions among the five groups (all *P* > 0.05) (Table 8). It was suggested that rt-PA or rPA combined with aspirin had lower adverse reactions in the treatment of hyper-acute CI.

**Table 8:** The rates of adverse reactions among the aspirin, rt-PA, rPA, rt-PA + aspirin, and rPA + aspirin groups

Types	Aspirin group (*n* = 117)	rt-PA group (*n* = 121)	rPA group (*n* = 123)	rt-PA + aspirin group (*n* = 126)	rPA + aspirin group (*n* = 124)	*P*
Gingival bleeding	12 (10.26)	11 (9.09)	10 (8.13)	3 (2.38)	2 (1.61)	0.009
Bleeding of the key parts (intracranial, digestive tract, respiratory tract, pericardium)	1 (0.85)	0 (0)	1 (0.81)	2 (1.59)	3 (2.42)	0.462
Bleeding of the other parts	0 (0)	2 (1.65)	3 (2.44)	5 (3.97)	4 (3.23)	0.286
Chills	1 (0.85)	0 (0)	2 (1.63)	4 (3.17)	2 (1.61)	0.323
Pain of double crura	2 (1.71)	1 (0.83)	2 (1.63)	5 (3.97)	4 (3.23)	0.451
Hypotension	1 (0.85)	4 (3.31)	2 (1.63)	3 (2.38)	5 (4.03)	0.515

Notes: rPA, Reteplase; rt-PA, Alteplase.

## Discussion

Acute CI is one of the most common diseases in clinical neurology [21]. The morbidity and mortality of acute CI are relatively high, which has become one of the main diseases endangering human health [22,23]. The occurrence of acute CI is due to the lack of blood supply to the brain tissue or the disorder caused by cerebral vascular flow instability and brain tissue in a long period of hypoxia, ischemia, and necrosis [24]. The main pathogenesis of large area CI is significantly related to the pathological changes of vascular wall, the change of blood components, and the abnormal hemodynamics [25]. Thrombolytic therapy has so far been the best intervention for AIS and has been shown to be a better known treatment for the only effective alternative to aspirin [26]. Rt-PA has been approved medical therapy as a treatment of AIS and is also suggested as the first-line treatment by most national and international stroke associations [27]. rPA is the agent of variants of tissue plasminogen activator, which the third generation of thrombolytic drugs with its advantages of convenience, fast, and good thrombolytic effect [28]. In this study, the NIHSS score, mRS score, and BI index, coagulation function, blood lipid changes, hemodynamics, safety evaluation, and adverse reactions were compared among the five groups, and we found that rt-PA or rPA combined with aspirin in the treatment of hyper-acute CI was better than those of rPA or rt-PA monotherapy.

Atherosclerosis was the main pathological basis of CI [29]. In the process of occurrence and development of CI, the body's blood lipids also changed significantly [30]. Because the abnormal increase of TC, TG, LDL-C, and decrease of HDL-C were related to atherosclerosis, they are often used as effective indexes for the prevention and evaluation of atherosclerosis [31]. In this study, TC, TG, LDL-C, and HDL-C were detected before and after treatment in each group, we found that the rt-PA + aspirin group and the rPA + aspirin group had better efficacy on improving the levels of TC, TG, LDL-C, and HDL-C.

There are obvious changes of coagulation function in patients with CI [32]. The coagulation mechanism of organism including endogenous coagulation pathway and the extrinsic coagulation pathway, and PT and APTT can reflect these two coagulation systems and common coagulation factor [33]. APTT is shorter, indicating that the coagulation function of the body is stronger and the blood is in a hypercoagulable state [34]. In addition to directly involving in the coagulation process, FIB can also mediate platelet aggregation, affecting the blood viscosity, which has high expression in patients with CI [35,36]. In this study, PT, APTT, and FIB were analyzed with the purpose of providing the reference and basis for a more reasonable and effective treatment plan in patients with acute CI. The result showed that in the rt-PA + aspirin and rPA + aspirin groups, the PT and APTT were prolonged, and the FIB decreased. The results indicated that the rt-PA + aspirin and rPA + aspirin groups had better efficacy in the intervention of acute CI. Furthermore, the clinical predictive validity of the NIHSS score has been shown in several investigations [37,38]. In addition, mRS and BI were also adopted in this study which made a further confirmation for this main result.

Intracerebral hemorrhage remains as the most feared side effects of thrombolytic therapy [39]. There are many reasons leading to intracerebral hemorrhage. It is believed that the history of diabetes or high blood glucose level, NIHSS score, CT imaging, 70 years of age or older and the use of standard dose have positive correlation with symptomatic cerebral hemorrhage [40,41]. In this study, 96 adverse reactions were recorded during 6 months of the follow-up period, but there had no difference among the five groups in terms of the rates of adverse reactions. In addition, in consistent with the former studies, hemorrhages were also common in this study.

In conclusion, rt-PA + aspirin and rPA + aspirin in the treatment of CI can improve the nerve injury of the patients, speed up the recovery of patients and reduce the incidence of adverse reactions, which is a safer and more effective treatment when compared with rPA or rt-PA monotherapy. The advantage is that we combined the multiple indicators to evaluate the efficacy and safety of the five therapies. However, because our analysis is based on relatively small sample sizes, we are cautious about extrapolating and generalizing our results. Thus, further researches are needed to do to confirm this view.

## Abbreviations

AIS: acute ischemic stroke
APTT: activated partial thromboplastin time
BI: Barthel Index
CI: cerebral infarction
CT: computerized tomographic
ESR: erythrocyte sedimentation rate
FIB: fibrinogen
HDL-C: high-density lipoprotein cholesterol
LDL-C: low-density lipoprotein cholesterol
mRS: modified rankin scale
NIHSS: National Institute of Health Stroke Scale
PT: prothrombin time
r-PA: recombinant plasminogen activator
rt-PA: recombinant tissue plasminogen activator
t-PA: tissue-type plasminogen activator
TC: total cholesterol
TG: triacylglycerol
